# Hepatic Venous Pressure Gradient Measurement in Bangladeshi Cirrhotic Patients: A Correlation with Child’s Status, Variceal Size, and Bleeding

**DOI:** 10.5005/jp-journals-10018-1235

**Published:** 2017-09-29

**Authors:** Mamun Al Mahtab, Sheikh M Noor E Alam, Mohammad A Rahim, Mohammad A Alam, Faiz A Khondaker, Ahmed L Moben, Masuda Mohsena, Sheikh Mohammad Fazle Akbar

**Affiliations:** 1 Department of Hepatology, Bangabandhu Sheikh Mujib Medical University, Shahbagh, Dhaka, Bangladesh; 2 Department of Hepatology Abdul Malek Ukil Medical College, Noakhali, Bangladesh; 3 Department of Hepatology, Dhaka Medical College, Dhaka, Bangladesh; 4 Department of Hepatology, Shaheed Suhrawardi Medical College, Dhaka, Bangladesh; 5 Department of Medicine, Kurmitola General Hospital, Dhaka, Bangladesh; 6 Department of Community Medicine, Ibrahim Medical College, Dhaka, Bangladesh; 7 Department of Medical Sciences, Toshiba General Hospital, Tokyo, Japan; 8 Department of Medical Sciences, Miyakawa Memorial Research Foundation Tokyo, Japan

**Keywords:** Child-Turcotte-Pugh, Hepatic venous pressure gradient, Portal pressure, Variceal bleeding.

## Abstract

**Background:**

Hepatic venous pressure gradient (HVPG) reflects the portal pressure in patients with cirrhotic portal hypertension. The aim of the study was to assess the relation of HVPG to variceal size, Child-Pugh status, and variceal bleeding.

**Materials and methods:**

A total of 96 patients with cirrhosis of liver were enrolled prospectively and each patient’s HVPG level was measured via the transfemoral route. Clinical and biochemical evaluation and upper gastrointestinal (GI) endoscopy were done in each subject. Severity of cirrhosis was assessed by Child’s status.

**Results:**

The mean HVPG was higher in patients with Child’s B and C (14.10 ± 7.56 and 13.64 ± 7.17 mm Hg respectively) compared with those of Child’s A (10.15 ± 5.63 mm Hg). The levels of HVPG differed significantly between Child’s classes A and B (p = 0.011) and Child’s A and C (p = 0.041). The mean HVPG was also higher in bleeders compared with nonbleeders with large varices (17.7 ± 5.5 *vs* 14.9 ± 4.7 mmHg respectively; p = 0.006).

**Conclusion:**

Hepatic venous pressure gradient seems to be important to assess the severity of liver cirrhosis.

**How to cite this article:** Al Mahtab M, Noor E Alam SM, Rahim MA, Alam MA, Khondaker FA, Moben AL, Mohsena M, Akbar SMF. Hepatic Venous Pressure Gradient Measurement in Bangladeshi Cirrhotic Patients: A Correlation with Child’s Status, Variceal Size, and Bleeding. Euroasian J Hepato-Gastroenterol 2017;7(2):142-145.

## INTRODUCTION

Portal hypertension is an inevitable consequence in the natural history of liver cirrhosis. It results from a combination of increased intrahepatic vascular resistance and increased blood flow through the portal venous system. Many complications including upper GI bleeding, hepatic encephalopathy, ascites, and renal dysfunction result from portal hypertension.^1^ Portosystemic collaterals develop when the portal pressure gradient increases to 10 mm Hg, whereas variceal bleeding occurs with a pressure gradient of more than 12 mm Hg.^2,3^

Because many of the complications in cirrhosis are due to increased portal pressure, it has been suggested that the assessment of portal hemodynamics could be helpful in predicting the course of cirrhosis. The direct measurement of portal pressure is an invasive and inconvenient procedure and associated with significant morbidity. Measurement of the HVPG which is the difference between wedged and free hepatic venous pressure (FHVP) is a simple, safe procedure, and it accurately reflects the portal pressure in patients with liver cirrhosis.^4-6^ The normal HVPG value lies between 1 and 5 mm Hg. Clinically significant portal hypertension is defined as HVPG ≥ 10 mm Hg.^6^ There is strong evidence that reduction of the HVPG to <12 mm Hg or by ≥20% of the baseline value significantly reduces the risk of recurrent variceal bleeding and mortality, making HVPG a valuable tool for the clinical management of cirrhosis.^7^

The impairment of liver function as determined by the Child-Turcotte-Pugh (CTP) score represents an important predictive factor for variceal hemorrhage.^8^ An improvement in Child’s score is associated with a decrease in HVPG.^9^ However, their interrelationship has not been properly explored.

There are sufficient data in the literature concerning the importance of portal pressure reduction for the primary and secondary prophylaxis of bleeding varices.^3^ However, clinical relevance and correlations of HVPG in several important aspects of chronic liver disease still remain to be fully studied. The relation of the rise in HVPG to the grade of varices and the severity of liver disease is also not well established, especially in a patient population with varying etiologies of cirrhosis.^9,10^ In fact, there is a paucity of large studies evaluating portal pressure parameters and seeking correlation with clinical and endoscopic variables of portal hypertension.

We conducted this study to assess if HVPG value in cirrhotic patients correlates with their Child’s status, size of varices, and variceal bleeding status in Bangladeshi patients.

## MATERIALS AND METHODS

Participants included 100 consecutive patients. They attended the Hepatology clinic of Farabi General Hospital, Dhaka, Bangladesh, and were diagnosed as patients of liver cirrhosis by clinical or biochemical, sonographic, and/or histologic findings. These patients were enrolled in the study from April 2015 to December 2016. Written informed consent was taken from all patients. A detailed clinical examination, baseline laboratory tests, endoscopy of upper GI tract was performed for all patients. Excluded from the study were patients presenting with underlying severe cardiac, respiratory, or psychiatric illness, hepatic encephalopathy, hepatocellular carcinoma, splenic or portal vein thrombosis, a prothrombin time index of <60%, or a platelet count of <50,000/cumm. Patients on therapy that may influence HVPG were excluded from the study. Hepatic venous pressure gradient measurements were done within 4 weeks of accomplishing endoscopy, and four patients were excluded from the study. Finally, the study was conducted in 96 patients with liver cirrhosis. This prospective study was approved by the institutional review board. Esophageal varices were graded as small (<5 mm) or large (>5 mm) according to the American Association for the Study of Liver Disease guidelines.^11^

## TECHNIQUE OF HVPG MEASUREMENT

Hepatic venous catheterization was done in the digital subtraction angiography suite (Siemens, Germany), and pressure was measured with a Sirecust 1260 (Siemens) strain gauge transducer. A 7F double-lumen balloon-tipped Swan-Ganz catheter was advanced into the right or middle hepatic vein through the percutaneous transfemoral route. With the balloon catheter advanced 2 cm into the right or middle hepatic vein, the FHVP was measured. With the continuous monitoring of pressure, the balloon was inflated by injecting air so as to wedge the hepatic vein and thereby measure the wedged hepatic venous pressure (WHVP). The wedged position was confirmed by gently injecting 2 mL of contrast agent through the catheter to demonstrate the retention of the contrast agent in the occluded portion of the hepatic vein. The HVPG level was calculated as the difference between the WHVP and FHVP readings.^12^

## STATISTICAL ANALYSIS

Statistical analysis was done using Statistical Package for the Social Sciences software (version 20.0; SPSS Inc., Chicago, Illinois, USA). Quantitative data were expressed as mean ± standard deviation and compared using Mann-Whitney and analysis of variance (ANOVA) tests. Correlation between variables was analyzed using Spearman’s correlation test. Statistical significance was defined as p ≤ 0.05.

## RESULTS

A total of 96 patients, in the age range of 18 to 72 years, were included in the study of whom 67 were males and 29 were females. The mean age of the study group was 45.74 years. In majority of the patients, cirrhosis of the liver was caused by chronic hepatitis B virus (HBV) infection (41.7%). Forty two (43.7%) of them had advanced Child’s class B or C liver disease, while rest 54 (56.3%) had Child’s class A. Ten (10.4%) had a history of previous variceal bleeding. Table 1 summarizes the demographic profile of the patients.

### HVPG and Child’s Class

**Graph 1:**
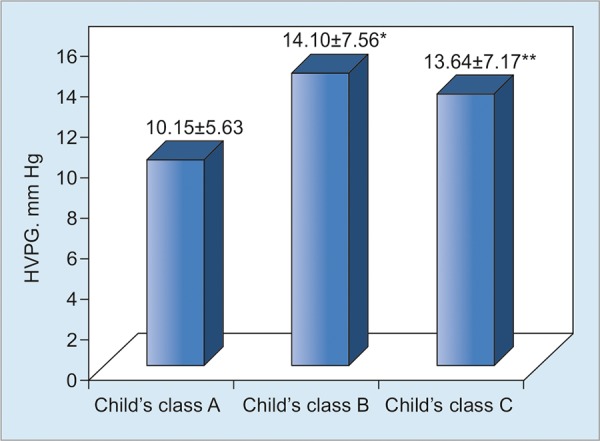
HVPG levels according to Child’s classes. *p = 0.011, comparing patients with Child’s A and B diseases, **p = 0.041, comparing patients with Child’s A and C diseases

The mean HVPG was higher in both Child’s B and C class patients (14.10 ± 7.56 and 13.64 ± 7.17 mm Hg respectively) compared with Child’s A class (10.15 ± 5.63 mm Hg). The difference between Child’s classes A and B, and Child’s classes A and C was found to be significant statistically (p = 0.011 and 0.041 respectively). However, the mean HVPG level of Child’s C was lower than that of Child’s B, but the difference was not statistically significant (p > 0.05; Graph 1).

**Table 1:** **Table 1:** Demographic profile of the study population

*Variables*		*No (%)*	
Total patients		96	
Male:Female		67:29	
Mean age (years)		45.74 ± 13.07	
Age range (years)		18-72	
*Etiology*			
HBV		51 (53.2)	
NASH		18 (18.8)	
Cryptogenic		16 (16.7)	
ALD		2 (2.1)	
Miscellaneous		9 (9.3)	
Total		96 (100)	
*Child’s classes*			
A		54 (56.3)	
B		20 (20.8)	
C		22 (22.9)	
*Varices*			
None		45 (46.9)	
Small		20 (20.8)	
Large		31 (32.3)	
*Variceal bleeding*			
Nonbleeders		86 (89.6)	
Bleeders		10 (10.4%)	

Child’s class: CTP class; ALD: Alcoholic liver disease; NASH: Nonalcoholic steatohepatitis

### HVPG and Variceal Size

**Graph 2:**
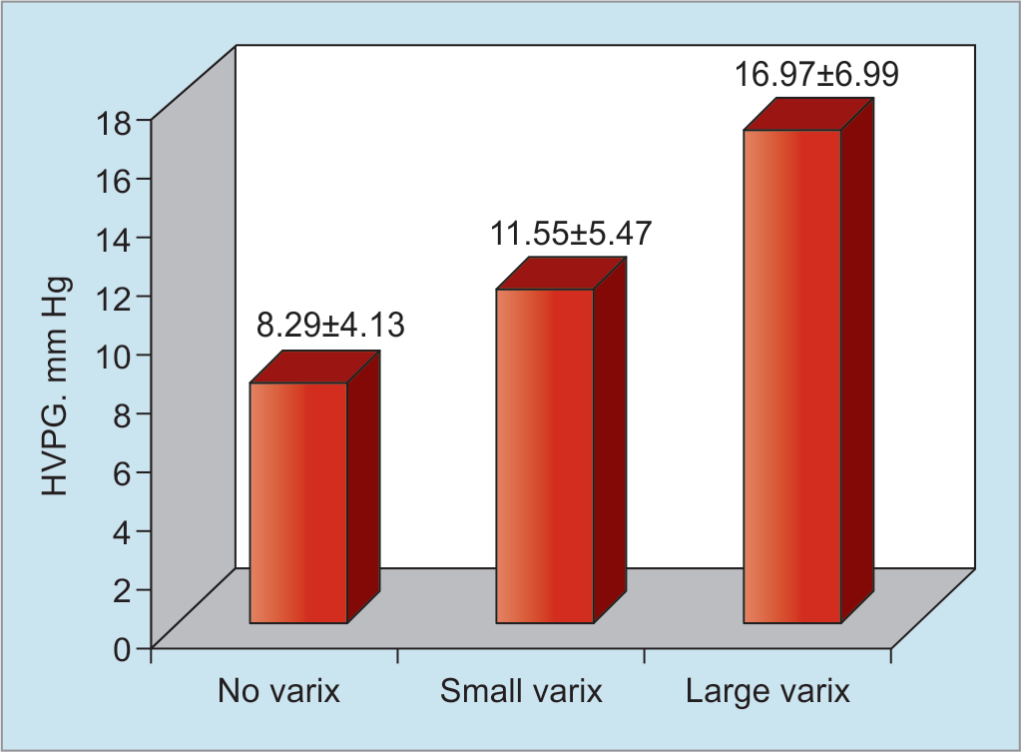
Mean HVPG levels (mm Hg) in patients with small, large, and no varices (p = 0.034 by one-way ANOVA test)

Esophageal varices were present in 51 out of 96 (53.1%) patients. Of the 51 patients with esophageal varices, 20 had small varices and 31 had large varices. The measured mean HVPG level was higher in patients with large varices (16.97 ± 6.99 mm Hg) than in patients with small varices (11.55 ± 5.47 mm Hg) and those without varices (8.29 ± 4.13 mm Hg; Graph 2). Overall, the difference among the three categories (small, large, and no varices) was statistically significant (p = 0.034). However, the difference between patients with small varices and those with large varices was not statistically significant (p = 0.54). When the mean HVPG level in patients with varices (14.84 ± 6.92) was compared with patients with no varices (8.29 ± 4.14), the difference was statistically significant (p = 0.009).

### HVPG and Bleeder Status

Out of the 96 patients, 10 (10.4%) had a history of previous variceal bleeding and all of them had large varices. Since nonbleeders had both small and large esophageal varices, patients with large varices were analyzed separately.

There were 10 (10.4%) variceal bleeders and 21 (21.87%) nonbleeders in the group of patients with large varices. The mean HVPG in bleeders *vs* nonbleeders with large varices was 17.7 ± 5.5 and 14.9 ± 4.7 mm Hg respectively (p = 0.006).

## DISCUSSION

We studied HVPG in 96 patients of HBV liver cirrhosis, nonalcoholic steatohepatitis related, cryptogenic, and miscellaneous etiology. Alcoholic cirrhosis comprised only two patients since this condition is rare in our population.

There was a strong correlation between HVPG level and CTP score in the study. Compared with the patients in CTP class A, the HVPG level was significantly higher among patients in CTP classes B and C. This finding indicates that the rise in HVPG correlates with the severity of liver disease and it is in uniformity with the results of two studies done in India.^13,14^ Although patients with Child’s C had significantly higher value than Child’s B patients in these studies, in our study Child’s C had a lower HVPG level (13.64 ± 7.17 mm Hg) than in patients of Child’s B (14.10 ± 7.56 mm Hg). However, the difference was not statistically significant.

Variceal bleeding is the most important cause of morbidity and mortality in cirrhotic patients. Almost half of the cirrhotic patients develop esophageal varices, with the lifetime prevalence around 80 to 90%.^15,16^ There are data showing that a reduction in HVPG decreases the size of varices.^17^ However, there is scarcity of data on the relation between variceal size and baseline HVPG. In this study, esophageal varices were present in 51 (53.1%) patients. The mean HVPG level in patients having varices (14.84 ± 6.92) was higher as compared with patients without varices (8.29 ± 4.14), the difference being statistically significant. The difference between small (11.55 ± 5.47 mm Hg) and large varices (16.97 ± 6.99 mm Hg) was found to be statistically insignificant. It could be a type II error. Similar correlation was found in the study done by Ramanathan et al.^14^

Controversy exists whether the mean HVPG is different between bleeders and nonbleeders. In our study, 10 (10.4%) patients were bleeders and 86 (89.6%) were nonbleeders. While all bleeders had large varices, non-bleeders had both small and large varices, and this may be considered as a confounding variable in the analysis. To eliminate this, HVPG levels in patients only with large varices with or without variceal bleeding were compared. The HVPG was significantly high in bleeders compared with nonbleeders, the difference being statistically significant (p = 0.006). These observations support the concept of a reduction in portal pressure to prevent the growth of varices and first variceal bleeding in patients with cirrhotic portal hypertension.^15^

In summary, this is the first report of its kind from Bangladesh. Like most of the previous studies, the results of this prospective study clearly emphasize the clinical relevance of measuring HVPG in patients with cirrhosis of the liver. The study shows that HVPG correlates with severity of liver disease, size of varices, and bleeder status. Patients with varices have a higher HVPG than patients with no varices, bleeders having a higher HVPG than nonbleeders.
